# Grafting of Polycaprolactone on Oxidized Nanocelluloses by Click Chemistry

**DOI:** 10.3390/nano3010141

**Published:** 2013-03-01

**Authors:** Abdelhaq Benkaddour, Khalil Jradi, Sylvain Robert, Claude Daneault

**Affiliations:** 1 Lignocellulosic Materials Research Center, University of Quebec at Trois-Rivières, 3351 des Forges avenue, Trois-Rivières, QC G9A-5H7, Canada; E-Mails: khalil.jradi@uqtr.ca (K.J.); sylvain.robert@uqtr.ca (S.R.); 2 Canada Research Chair on Value-Added papers, Trois-Rivières, QC G9A-5H7, Canada; E-Mail: claude.daneault@uqtr.ca

**Keywords:** oxidized nanocelluloses, polycaprolactone, click chemistry, composites, grafting

## Abstract

The main objective of this work is the grafting of polycaprolactone diol (PCL) on the surface of oxidized nanocelluloses (ONC) in order to enhance the compatibility between the hydrophilic cellulose nanofibres and the hydrophobic polymer matrix. This grafting was successfully realized with a new strategy known as click chemistry. In this context, the oxidized nanocelluloses bearing alkyl groups (ONC-PR) were prepared by reacting amino groups of propargylamine (PR) with carboxyl groups of ONC. In parallel, PCL was converted into azido-polycaprolactone (PCL-N_3_) in two steps: (i) tosylation of polycaprolactone (PCL-OTs) and (ii) conversion of PCL-OTs into PCL-N_3_ by nucleophilic displacement using sodium azide. Finally, ONC-PR was reacted with PCL-N_3_ in heterogeneous conditions through click chemistry in order to prepare polycaprolactone grafted oxidized nanocellulose (ONC-g-PCL), which could be suitable for improving the interfacial adhesion in the composite materials. The grafted samples were characterized by transmission electron microscopy and by Fourier transform infrared spectroscopy (FTIR), X-ray photoelectron spectroscopy (XPS) and Carbon-13 nuclear magnetic resonance spectroscopy (^13^C-NMR) spectroscopic techniques.

## 1. Introduction

During the last few decades, bio and nanomaterials have gained an increasing interest as alternatives to decrease the dependency on petroleum-based products. Among these biomaterials, cellulose is the most abundant organic raw material, renewable and biodegradable resource and finds applications in different areas, especially as reinforcement in such biocomposites. One of these applications has been the production and development of nanocelluloses, which are potential nanosized reinforcements. Cellulose is part of an architectural edifice complex, which varies with the organism. The individual cellulose chains are associated by hydrogen bonds into microfibrils having diameters between 2 and 20 nm and lengths ranging from 100 nm to several micrometers, depending on their biological origin [1].

Referring to the literature, different types of cellulose microfibrils have been produced by other physical and chemical treatments of cellulose fibres [2,3,4,5,6,7,8]: mechanical treatment, acid hydrolysis and catalyzed oxidation. In the case of mechanical treatment, the resulting products consist mainly of bundles of microfibrils, and it requires a large amount of energy. Thus, it has not been sufficient to individualize cellulose microfibrils using only mechanical treatment. Another route toward the preparation of nanocelluloses relies on acid treatment. This consists of dissolving the amorphous domains, leading to the formation of cellulose nanocrystals having dimensions, which depend on the type of acid, acid concentration, time, temperature of hydrolysis reaction and the different origins of cellulose [3,7,8,9,10,11,12]. One of the main disadvantages of this hydrolytic treatment is the depolymerization of cellulose chains that promotes a dramatic decrease in the microfibril length and width, which negatively affects the structure of microfibrils [1,8,9,10,11,12,13]. To overcome these problems, catalyzed oxidation is an alternate promising route to obtain individualized microfibrils. This technique is based on the conversion of alcohol groups of cellulose into carboxylic acids using 2,2,6,6-tetramethyl-1-piperidine oxoammonium radical (TEMPO) in the presence of sodium hypochlorite and sodium bromide. With such reagents, the oxidation is selective, as it oxidizes exclusively the primary hydroxyl groups, while leaving untouched the secondary ones [2]. The chemical modification of these cellulosic substrates provides a versatile route, which allows us to introduce other functionalities for developing new materials. The reason of using ONC as a reinforcement agent in the composite materials is that ONC are considered to be cellulosic nanomaterials (5–20 nm wide single-sized microfibrils), which are composed of crystalline and amorphous domains that are generated by the TEMPO oxidation process [14].

In this context, the oxidized cellulose-containing carboxyl and hydroxyl groups can serve as templates for grafting other molecules of interest, thus opening new horizons for many applications. Esterification and etherification are the most common approaches for modification reactions of oxidized cellulosic fibres. Despite all, there are some limitations concerning the use of this cellulosic nanomaterial. In fact, the strong hydrophilic behaviour oxidized cellulose (COOH and OH) has a tendency to form hydrogen bonds between adjacent fibrils and reduces the interaction with the hydrophobic polymer matrix [15]. In addition, the use of nanofibres has been mostly restricted to water soluble polymers. For this reason, it is necessary to reduce the entanglement of the nanofibres and improve their dispersion in the hydrophobic matrix by surface modification of nanofibres without deteriorating their reinforcing capability.

To overcome this problem, chemical and physical surface modification of different cellulose fibres has been accomplished using low-molecular-weight compounds and polymers [16,17,18]. One effective method for chemical modification of cellulose substrates is to covalently bond a hydrophobic polymer onto the surface of oxidized cellulose fibres by using a new strategy known as click chemistry. Since 2002, click chemistry has conquered the synthesis world. This chemical philosophy, with efficient, selective and versatile reactions [19], has given new opportunities, particularly in creating new composite materials. The Cu (I)-catalyzed variant of the Huisgen 1,3-dipolar cycloaddition between terminal alkynes and azides is the most famous click reaction. It has been widely used in the synthesis of functionalized polymers, new monomers/macromonomers and block copolymers [20,21]. This new concept allows one to greatly expand the diversity of structures of polysaccharides, since it helps produce compounds that are not accessible via etherification, esterification and the most commonly applied reactions [22,23]. As this method is very efficient and environmentally clean [24], we decided to use it in the final step of synthesis in the present work. Recently, polyesters have been considered as the most versatile group of degradable polymers; they have been used in different areas, such as biomedical applications, bulk applications, such as packaging [25,26], and bionanocomposite materials with significant improvements in terms of mechanical performances [27].

The aim of this work is the modification of oxidized nanocelluloses (ONC) by attaching hydrophobic polymers, such as polycaprolactone (PCL), via click chemistry in order to improve the compatibility between the PCL matrix and ONC to produce new bio-nanocomposites.

PCL has already been used as a grafting molecule on different cellulosic materials. For example, Habibi *et al.* [28,29] have grafted PCL with various molecular weights via isocyanate-mediated reaction on the surface of cellulose nanocrystals obtained by acid hydrolysis. Further, the “grafting from” approach was used to graft poly(ε-caprolactone) polymers to cellulose nanocrystals and microfibrillated cellulose by Sn(Oct)_2_-catalyzed ring-opening polymerization [18,30]. With such a method, PCL could be grafted with high molecular weight. While in the case of the “grafting to” approach, PCL (*M*w = 80,000 or more) can not diffuse into the fibre, because of its large molecular weight, and only surface grafting may occur. It should be emphasized that, because of the large molecular weight of PCL, which induces an important steric hindrance and, thus, prevents the grafting reaction, we decided to graft ONC using a PCL with low-molecular-weight (*M*_W_ = 2400).

To the best of our knowledge, no study has been made so far to graft PCL onto Tempo-oxidized nanocellulose. Further, the click reaction has been widely described in the literature for a homogeneous systems [19], and there are only two reports on its use for heterogeneous systems [19,31]. In the present work, we have grafted PCL onto oxidized nanocelluloses using click chemistry. Initially, the primary hydroxyl groups on the surfaces of bleached kraft pulp were selectively activated to carboxylic acids using TEMPO-mediated hypohalite oxidation. Then, propargylamine was grafted onto the surface of activated ONC; this coupling (ONC-PR) is a nucleophilic reaction between the amine group (–NH_2_) of propargylamine and the carboxyl group (–COOH) of the ONC using hydrochloride-1-ethyl-3 (3dimethylaminopropyl) carbodiimide (EDAC) in the presence of an activation agent, such as *N*-hydroxysuccinimide (NHS) [32]. In parallel, polycaprolactone-diol (PCL) was converted to azido derivative (PCL-N_3_); this was achieved in two steps. First, PCL-diol is tosylated by para-Toluenesulfonyl chloride, which is a very good leaving group, and then tosylated PCL is converted into azido-polycaprolactone by nucleophilic displacement using sodium azide. In the last step, alkyne (ONC-PR) and azide (PCL-N3) were brought together via click chemistry.

## 2. Results and Discussion

### 2.1. TEMPO-Mediated Oxidation of Cellulose

**Figure 1:**
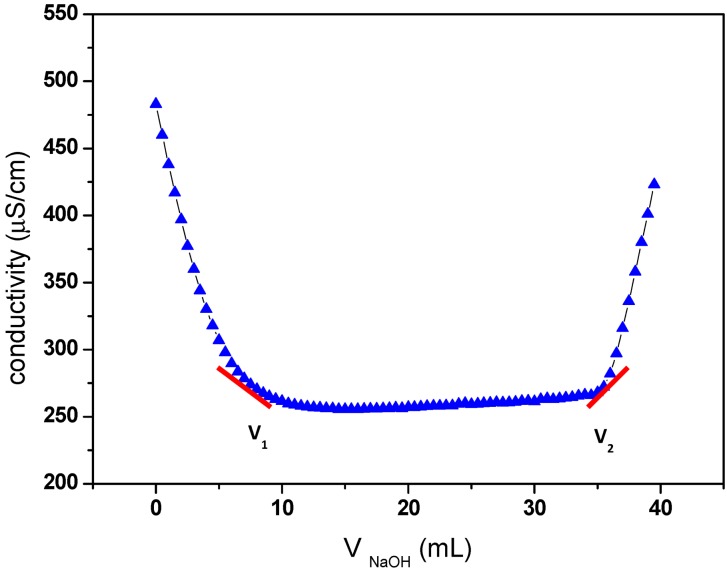
Electric conductivity titration of the oxidized cellulose.

The carboxyl content of oxidized cellulose samples was determined by the electric conductivity titration method [5], as described in Materials and Methods. The results of this titration are shown in Figure 1, and a carboxylic content of 1.17 was obtained.

The degree of oxidation (DO) was equal to 0.25, and it is given by the following equation [33]:

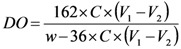

where *C* is the NaOH concentration (mol/L), *V*_1_ and *V*_2_ are the volume of NaOH (in Litre) required to neutralize the excess protons of hydrochloric acid and those attached to the carboxylic groups, respectively, *w* is the weight of the oven-dried sample (g), 36 is the difference between the molecular weight of the anhydroglucose unit and that of the sodium salt of a glucuronic acid and 162 is the molecular weight of one anhydroglucose unit. The grafting density of propargylamine was calculated based on the *DO* value (0.25) of oxidized cellulose determined by conductivity titration. The value of 0.25 means that 25% of the hydroxymethyl groups on cellulose have been oxidized to corresponding carboxylic acid. Otherwise, every fourth of the anhydroglucose units in ONC contain one carboxyl group. So, the maximum grafting density of propargylamine corresponds to the situation where every fourth of the anhydroglucose units in ONC contain a grafted propargylamine moiety (when the totality of carboxylic acids react with propargylamine). As a result, the maximum amount of nitrogen grafted in on oxidized nanocelluloses should be equal to 2.03% (theoretical value = 14/[4 × (162+40)], where 14, 162 and 40 are the molecular weight of nitrogen, the anhydroglucose unit and the attached part of propargylamine, respectively). However, the amount of nitrogen found in the ONC-PR as obtained by X-ray photoelectron spectroscopy (XPS) was 1.88% (experimental value/theoretical value ≈ 1.88/2.03. See XPS results). This corresponds to a grafting density of ≈ 92%.

### 2.2. FTIR Experiments

**Figure 2:**
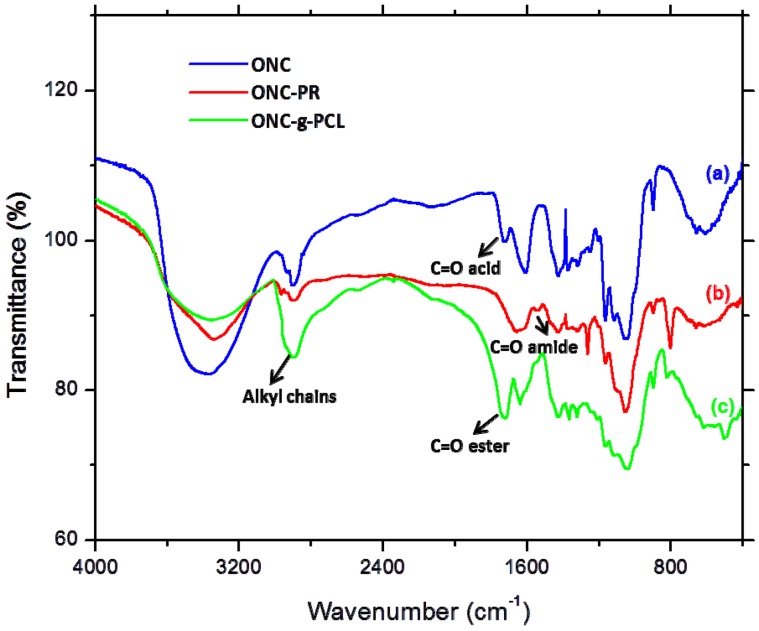
Fourier transform infrared spectroscopy (FTIR) spectra of oxidized nanocelluloses (ONC) alone (**a**), ONC-propargylamine (PR) (**b**) and ONC-g-polycaprolactone (PCL) (**c**).

Figure 2 shows the Fourier transform infrared spectroscopy (FTIR) spectra of ONC and ONC derivatives. Spectrum a in Figure 2 shows one peak near 1734 cm^−1^ corresponding to the C=O band of the carboxylic acid of ONC, which confirms the oxidation of primary alcohols of native cellulose [34]. The second at 1615 cm^−1^ presents the OH bending of absorbed water [35]. The disappearance of the carbonyl stretching band after grafting the propargylamine on ONC (spectrum b) is due to the formation of the amide linkages between the acid groups of ONC and the amino groups of propargylamine. The FTIR band of the C–N bond of the amide appears as a small shoulder at 1534 cm^−1^. Finally, the grafting efficiency of polycaprolactone onto the surface of ONC was confirmed by the appearance of a carbonyl stretching band of polyester at 1741 cm^−1^ and an increase in band intensity of an alkyl chain at 2900 cm^−1^ (spectrum c).

**Figure 3:**
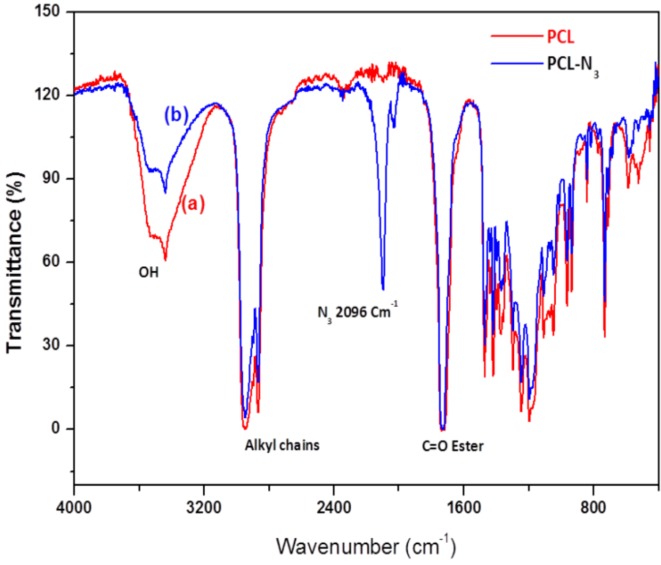
FTIR spectra of PCL (**a**) and azido-polycaprolactone (PCL-N_3_) (**b**).

Figure 3 shows the FTIR spectra of PCL and PCL-N_3_. The spectrum of PCL (Figure 3a) shows a peak around 3500 cm^−1^ and a very sharp signal at 1750 cm^−1^, corresponding to hydroxyl and ester groups, respectively. When tosylated polycaprolactone reacted with NaN_3_, the FTIR spectrum of PCL-N_3_ (Figure 3b) shows a considerable decrease in the intensity of the peak at 3500 cm^−1^ corresponding to OH groups and the appearance of a new intense band at 2096 cm^−1^ typical of the azide groups, which confirms clearly that more and more azide molecules are covalently coupled to the surface of PCL chains [31].

### 2.3. TEM Images

**Figure 4:**
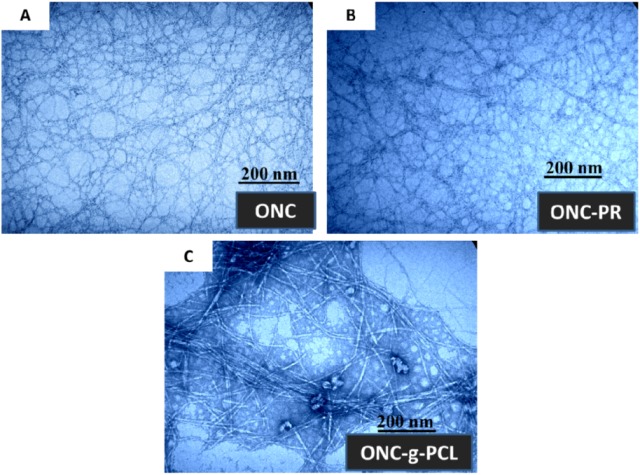
Transmission electron micrographs of: (**A**) ONC, (**B**) ONC-PR and (**C**) ONC-g-PCL.

The Transmission electron micrographs (TEM) images of ONC, ONC-PR and ONC-g-PCL are displayed in Figure 4. Figure 4A shows that ONC fibres are individualized with a width of about 3–4 nm and length exceeding 1 µm. Shape and size of individualized ONC are similar to those reported by other researchers [6,14]. After grafting ONC with propargylamine (Figure 4B), we do not notice a significant change in the morphology of ONC. Using image processing software, the TEM image of ONC-g-PCL (Figure 4C) reveals a significant increase in the width of grafted ONC fibres (25–30 nm). The increase of the width of the grafted ONC fibrils confirms clearly that the PCL chains are incorporated onto the surface of the ONC network.

### 2.4. XPS Results

**Figure 5:**
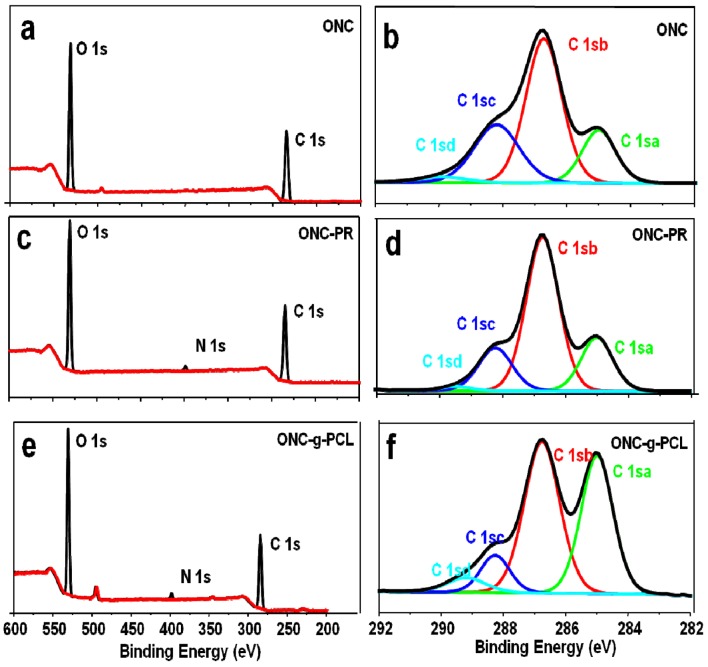
X-ray photoelectron spectroscopy (XPS) spectrum of ONC (**a**), ONC-PR (**c**), ONC-g-PCL (**e**) and deconvolution of their respective C_1s_ peaks (**b**), (**d**) and (**f**).

To further confirm the grafting of PCL on ONC, we have studied our samples using X-ray photoelectron spectroscopy. This technique presents the advantage of determining the composition of a surface layer with a thickness of approximately 10 nm, whereas FTIR spectroscopy presents a depth of analysis of several micrometers. Figure 5 shows the XPS spectra of ONC, ONC-PR and ONC-g-PCL. The carbon composition and the experimental atomic composition as determined from the XPS spectra analysis and the calculated oxygen to carbon (O/C) ratio for all samples are summarized in Table 1, Table 2, respectively. All XPS spectra reveal that C and O are the predominant species and they occur at 285 and 532 eV, respectively. The grafted samples show a new peak at 400 eV corresponding to the N atom. The deconvolution of the peak corresponding to carbon atoms in the C 1s XPS spectrum shows four types of carbon bonds: C–C/C–H (C 1sa, 285 eV), C-O (C 1sb, 286.7–8 eV), C=O (C 1sc, 288.2–3 eV) and O–C=O (C 1sd, 289.2–9 eV). The intensity of the C 1sa peak increased from 18.14% for ONC, as reference to 38.59% for ONC-g-PCL (Table 1), which is the consequence of the presence of the alkyl chain of the polymer. Additionally, the intensity of the C 1sd peak decreased from 2.49% for ONC to 1.32% for ONC-PR. This decrease was due to the formation of an amide bond between the acid group of ONC and the amino group of the propargylamine. The same peak (C 1sd) increased from 1.32% for ONC-PR to 5.1% for ONC-g-PCL, which confirms the grafting of the polyester chain on the ONC [27,31,36]. Finally, the analysis of data presented in Table 2 shows that the O/C atomic ratio of ONC decreased (from 0.58 to 0.37) as a result of PCL grafting on ONC.

**Table 1:** C 1s narrow scan XPS spectra for ONC, ONC-PR and ONC-g-PCL.

Binding type	C 1sa (C–C/C–H)	C 1sb (C–O)	C 1sc (C=O)	C 1sd (O–C=O)
Energy (eV)	285	286.7–8	288.2–3	289.2–9
ONC	18.14	52.71	26.67	2.49
ONC-PR	20.62	60.66	17.40	1.32
ONC-g-PCL	38.59	46.77	9.55	5.1

**Table 2:** Experimental atomic composition and O/C ratio obtained by XPS analysis for ONC, ONC-PR and ONC-g-PCL.

Sample	Atomic content %			O/C
	C	O	N	
ONC	63.16	36.84	0	0.58
ONC-PR	63.24	34.88	1.88	0.55
ONC-g-PCL	70.19	26.54	2.35	0.37

### 2.5. ^13^C-NMR Results

**Figure 6:**
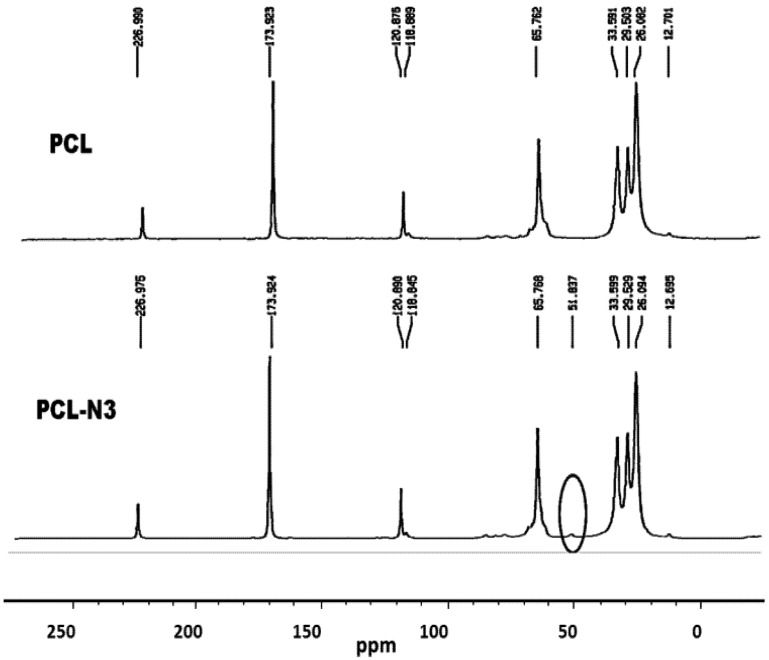
^13^C-nuclear magnetic resonance (NMR) spectra of PCL and PCL-N_3_.

**Figure 7:**
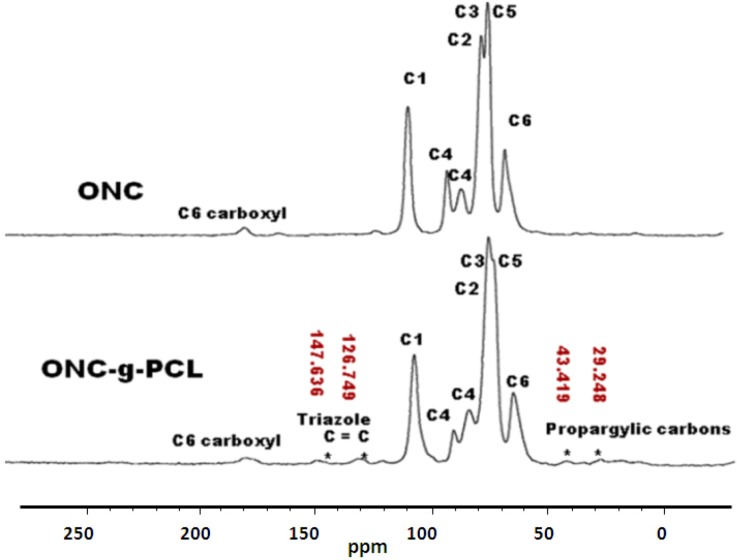
^13^C-NMR spectra of ONC and ONC-g-PCL.

To verify a successful click reaction (formation of triazole moiety), we have further characterized our samples using the ^13^C-NMR technique. ^13^C-NMR Spectra of ONC, PCL and their derivatives are shown in Figure 6, Figure 7.

The results obtained by ^13^C-NMR analysis are in agreement with those obtained by XPS and FTIR. Figure 6 shows clearly the appearance of the peak at 51.8 ppm assigned to the terminal methylene moiety linked to the azido group [31]. In the case of ONC and ONC-g-PCL, Figure 7 (ONC-g-PCL) shows the appearance of two peaks at 126 and 147 ppm for the triazole ring [37,38] and two others peaks at 29 and 43 ppm for propargylic carbons [38]. However, the PCL methylene carbons in the case of ONC-g-PCL are not detected (weak signals between 20 and 30 ppm); this is not totally surprising, because the reactions were carried out under heterogeneous reaction conditions. In addition, the used PCL (oligomer) has a low-molecular-weight (Mn = 2000, Dpn = 20), while oxidized nanocelluloses have a high molecular weight (Mn ~ 40,500, Dpn ~ 250). The reaction yield was found to be around 14%, as determined by gravimetry (low). This weakness is due to the strong hydrophilic behaviour of oxidized cellulose, which has a tendency to form hydrogen bonds between adjacent fibrils and to reduce the interaction with PCL. Efforts to further control the uniformity of this grafting reaction are underway in our laboratory by appropriately adjusting the grafting conditions.

## 3. Experimental Section

### 3.1. Materials

A Commercial never-dried bleached kraft pulp was used as the starting material for the oxidized nanocelluloses, propargylamine (98%), *N*-ethyl-*N'*-(3-dimethylaminopropyl) carbodiimide hydrochloride (EDAC.HCl), *N*-hydroxysuccinimide (NHS), 2-(*N-*morpholino)-ethanesulfonic acid (MES), polycaprolactone-diol (Mn 2000; Sigma-Aldrich), p-toluenesulfonyl chloride (TsCl), sodium azide (NaN_3_), sodium L-ascorbate and copper (II) sulphate pentahydrate (CuSO_4_(H_2_O)_5_) were purchased from Sigma-Aldrich. Chemicals and solvents were commercial products used as received.

### 3.2. Methods

#### 3.2.1. Preparation of Oxidized Nanocelluloses ONC

According to the method of Saito *et al*. [6], 4 g of never-dried kraft pulp was suspended in water containing 0.2 g of TEMPO and 1.2 g of sodium bromide. The reaction was initiated by adding 25 mL of 13% NaClO at room temperature under gentle agitation. The reaction pH was maintained at 10.5 by adding 0.5 M NaOH. When no more decrease in pH was observed, the reaction was stopped by the addition of methanol (40 mL), and the pH was adjusted to 7 by adding 0.5 M HCl. The oxidized pulp was filtrated and washed with de-ionized water.

#### 3.2.2. Measurement of Carboxyl Group Content

The carboxylic content in the oxidized cellulose was determined using the conductometric titration method using a Dosimat 765 (Metrohm) titrator according to the technique of Saito and Katz [5,28]. In this procedure, the sodium carboxylate groups in the TEMPO oxidized celluloses were converted to the free carboxyl form by treating the sample with 0.1 M HCl solution three times and, finally, thoroughly washed with de-ionized water to remove the excess acid. The oxidized pulp prepared in this way was transferred to a 600 mL beaker containing 450 mL of 0.001 N NaCl solution and mixed well. Five millilitres of 0.1 N HCl was added to the fibre suspension before starting titration of the carboxylate groups with 0.1 N NaOH solution. At the end of the titration, the fibres were filtered, washed and dried in an oven at 105 °C to determine the exact weight of the sample. The carboxyl content expressed in mmol/g was calculated by the software.

#### 3.2.3. Production of Oxidized Nanocelluloses (ONC)

Zero-point-four grams of oxidized cellulose were suspended in 400 mL of water (0.1%). The slurry was mechanically homogenized with a domestic blender for 20 min [24]. The obtained suspension was centrifuged at 10,000× *g* for 15 min in order to separate oxidized nanocelluloses (supernatant) from the infibrillated fractions.

#### 3.2.4. Synthesis of Click Precursor Bearing Alkyne Groups (ONC-PR)

**Scheme 1:**
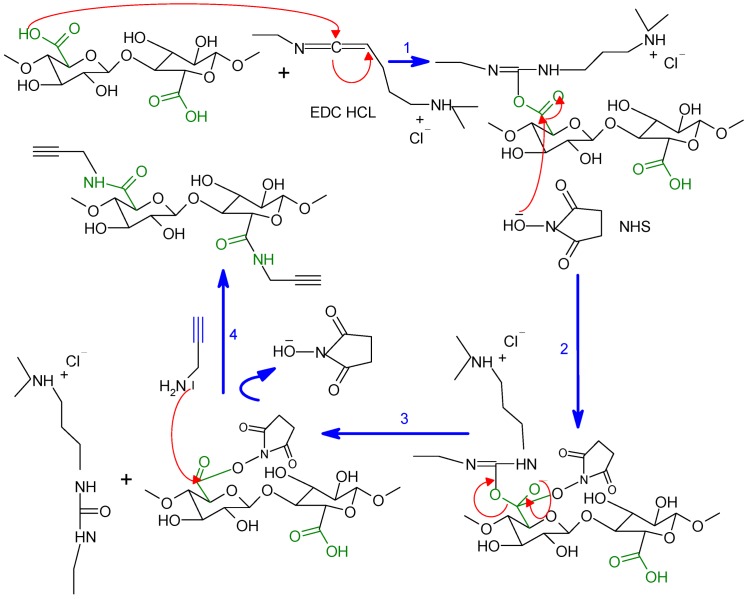
The coupling mechanism between oxidized nanocellulose (ONC) and propargylamine.

Among the many methods employed to obtain an amide linkage from carboxylic acids and amine groups, we chose that calling upon the use of the EDC/*N*-Hydroxysuccinimide (NHS) as the activating system, as described in Scheme 1. One-point-two-five millilitres of the ONC suspension 0.2% (~50 mg of dried ONC) were added to 6 mL of MES [2-(*N*-morpholino)-ethanesulfonic acid buffer (50 mM, pH = 4)] under magnetic stirring. Then, 120 mg EDC·HCl [*N*-(3-dimethylaminopropyl)-*N'*-ethylcarbodiimide hydrochloride)], 72 mg NHS (*N*-Hydroxysuccinimide) and 60 μL of propargylamine, respectively, were added to the ONC suspension, and the mixture was continually stirred at room temperature for 24 h. The suspension was then dialyzed (cutoff = 12 kDa) against a saturated NaCl for 1 day and then against distilled water for 3 days. Finally, the nanocellulose precursor (ONC-PR) was recovered by freeze-drying.

#### 3.2.5. Conversion of Polycaprolactone into Azido-Polycaprolactone (PCL-N_3_)

**Scheme 2:**
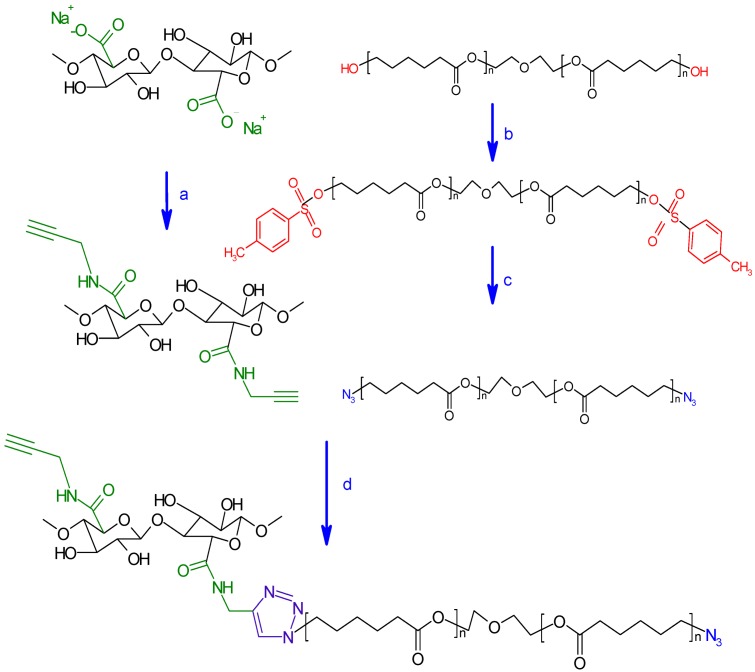
Strategy of oxidized nanocelluloses modification adopted in this work. (**a**) propargylamine, MES Buffer pH 4, EDC/NHS, R.T., 24 h; (**b**) TsCl, THF, triethylamine, triethylamine hydrochloride, R.T., 24 h; (**c**) DMF, NaN_3_, R.T., 24 h; (**d**) THF, sodium ascorbate, CuSO_4_, 5H_2_O, R.T., 48 h.

The azidation of polycaprolactone-diol into azido-polycaprolactone was achieved in two steps as proposed by Krouit *et al*. [22] (Scheme 2). In the first step, 11.44 g of TsCl (60 mmol, 5 equiv/PCL-diol) were dissolved in 40 mL of THF and added dropwise to a stirred solution containing PCL (24 g, 12 mmol), Et_3_N (41.5 mL, 0.3 mol, 25 equiv/PCL-diol), trimethylamine hydrochloride (573 mg, 6 mmol, 0.1 equiv/TsCl) in 40 mL of tetrahydrofuran (THF) at room temperature. The mixture was stirred for 1 day. Insoluble products were filtered out, and the clear reaction mixture was poured into a bath of ethyl ether at 0 °C. The precipitated product, p-toluenesulfonyl-polycaprolactone (PCL-OTs), was recovered and dried under vacuum. The prepared polymer was then weighted, and a yield of 70% was determined. In the second step, 1 g of sodium azide was added to a solution of PCL-OTs (15 g) in 40 mL of dimethylformamide (DMF) under moderate stirring at room temperature for 24 h. The mixture was then filtrated to remove insoluble products, and the ensuing filtrate was poured into hexane at 0 °C. The precipitated azido-polycaprolactone (PCL-N3) was recovered and dried under vacuum. The reaction yield was about 80%.

#### 3.2.6. Grafting of Azido-Polycaprolactone onto ONC-PR by Click Chemistry

One-hundred and three grams of ONC-PR and 192 mg PCL-N3 were mixed in 20 mL of THF. Next, 7.5% *w*/*v* of CuSO_4_ 5H_2_O aqueous solution (100 μL, 0.03 mmol) and 120 μL of ascorbic acid (1 M sol, 0.12 mmol) were added. The heterogeneous mixture was stirred in the absence of light at room temperature. After a 48 h reaction, the grafted ONC (ONC-g-PCL) were filtered and washed with CH_2_Cl_2_ and water. After successive Soxhlet extraction with methylene chloride and water, the sample was recovered and dried at 50 °C for 48 h before being characterized.

### 3.3. Instrumentation

#### 3.3.1. Fourier Transform Infrared Spectrometry (FTIR)

Two-percent w/w of dried sample was mixed with KBr, and pellets of the mixture were made. FTIR spectra were recorded using a Perkin-Elmer System 2000 in transmission mode. A total of 32 scans were taken per sample with a resolution of 4 cm^−1^ (4000–400 cm^−1^).

#### 3.3.2. X-Ray Photoelectron Spectroscopy (XPS)

XPS experiment was carried out using a Kratos Axis Ultra spectrometer equipped with a monochromatic Al Kα X-ray source (E = 1486.6 eV) with a power of 225 W. Samples were placed in an ultrahigh vacuum chamber (10^−9^ torr at room temperature) with electron collection by a hemispherical analyzer at a 90° angle. The overall spectrum was shifted to ensure that the C–C/C–H contribution to the C1s signal occurred at 285.0 kV. Gaussian peak profiles were used for spectral deconvolution of C1s spectra.

#### 3.3.3. Transmission Electron Microscopy (TEM)

Drops of the suspensions were deposited onto glow-discharged carbon-coated electron microscopy grids. The excess liquid was absorbed by a piece of filter paper, and a drop of 2% uranyl acetate negative stain was added before drying. The liquid in excess was wiped off, and the remaining film of stain was allowed to dry. The specimens were observed using a Philips EM 208S microscope operating at 80 kV.

#### 3.3.4. ^13^C-NMR

Solid-state CP/MAS ^13^C spectra were recorded on a Bruker Advance Spectrometer (Bruker Biospin Inc, Milton, ON, Canada) at 75 or 100 MHz (magnetic fields of 7.0 or 9.4 Tesla). Samples were spun at 6 to 8 kHz in a 4 mm ZrO_2_ rotor at the magic angle and at room temperature. A contact time of 1 ms and a relaxation time of 1s were accorded to an acquisition of 3000 transients to produce a good quality spectrum. A high power phase altered proton decoupling time-proportional-phase-increment (TAPPI) was applied during free induction decay (FID) acquisition. ^13^C chemical shifts were determined with reference to adamantine.

## 4. Conclusions

We have successfully grafted the polycaprolactone onto oxidized nanocellulose in heterogeneous medium using a click chemistry reaction. This approach represents a rapid, convenient and versatile synthesis of grafted cellulosic nanofibres in mild conditions. This surface modification of oxidized nanocellulose was successfully achieved in four steps. The obtained PCL-grafted ONC has been characterized using TEM and FTIR, XPS and ^13^C-NMR spectroscopies. The results of these techniques confirm this grafting. Consequently, the modification of ONC with hydrophobic polymers, such as PCL, reduces their hydrophilic character by blocking surface hydroxyl and carboxyl groups and, thus, could reduce the moisture uptake. Work is in progress in order to study the mechanical properties and moisture absorption of the obtained product.
